# Azobenzene protonation as a tool for temperature sensing

**DOI:** 10.3762/bjoc.21.115

**Published:** 2025-07-28

**Authors:** Antti Siiskonen, Sami Vesamäki, Arri Priimagi

**Affiliations:** 1 Smart Photonic Materials, Faculty of Engineering and Natural Sciences, Tampere University, Korkeakoulunkatu 3, FI-33720 Tampere, Finland

**Keywords:** azobenzene, protonation, sensing, spectral changes, temperature

## Abstract

Azobenzenes can be protonated at one of the azo nitrogen atoms, leading to significant changes in their absorption and photochemical properties. While azobenzene protonation has been recently used as a tool in photoswitching studies, the factors influencing protonation itself have received little attention. Here, we report a strong temperature dependence of azobenzene protonation in 1,2-dichloroethane and highlight its potential for temperature sensing applications. Density functional theory calculations were performed to support our findings and to investigate the mechanisms of azobenzene–acid interactions, aiming to guide the design of azobenzene-based temperature sensors in future research.

## Introduction

Molecular switches are molecules that can reversibly shift between distinct (meta)stable states in response to external stimuli such as light, pH changes, or electric fields [1]. Over the past decades, they have emerged as staple building blocks in smart materials, from light-responsive norbornadienes in molecular solar thermal energy storage [2] to pH-sensitive spiropyrans in cell imaging [3], and redox-active viologens in memory junctions [4]. Among the different molecular switches azobenzenes stand out due to their widespread use in biomedicine [5–6], energy storage [7–8], soft robotics [9–10], and sensing [11]. The key factors for their success are the efficient photochemical isomerization, causing large spatial, spectral, and electronic changes, as well as the relative ease of modification of the azobenzene core by introducing different functional groups to the phenyl rings. For these reasons, azobenzenes can be sensitized to various stimuli and easily integrated into different types of materials.

While *cis*–*trans* isomerization is the most prominent property of interest for azobenzenes, there are also other transformations that can offer useful functionalities. These include ionization via protonation of one of the nitrogens forming the azo bond [12–13], azo–hydrazone tautomerization [14–15], and binding of analytes to side groups [16]. Such transformations depend on the azobenzene structure but they all typically result in significant changes in the absorption spectrum and/or alter the stability of the metastable isomer. In sensing, azobenzenes have been utilized, e.g., through changes in isomerization kinetics [17–18] or colorimetric properties. The colorimetric sensors are effective for heavy metal detection [16,19] and humidity sensing [20]. The color changes can be either reversible or irreversible, depending on the mechanism of operation. Spectral tuning also enables switching with low-energy light, eliminating the need for potentially harmful UV irradiation [21].

Utilizing spectral changes caused by azobenzene protonation is a well-established concept, with methyl orange-based pH indicators dating back to the late 1800s [22]. Early studies primarily focused on characterizing these spectral changes, determining p*K*_a_ values, and elucidating the protonation mechanism of different azo compounds [23–25]. More recent work has harnessed protonation to enable red-light switching [12–13,26] and to control the stability of the *cis*-isomer [27–29]. These studies have focused on how protonation affects isomerization, with less attention given on how external stimuli affect the protonation itself.

In this study, we investigate the temperature dependence of azobenzene protonation with the aim of utilizing it in temperature sensing. We focus on steady-state behavior of azobenzene, 4-methoxyazobenzene, and 4,4’-dimethoxyazobenzene in solution. By tuning acid strength and solvent polarity, we demonstrate that the degree of protonation, and thus solution color, is highly temperature-dependent, changing from pale yellow to deep red as temperature decreases. We also present mechanistic insights and compare our experimental findings with computational results.

## Results

**Figure 1:**
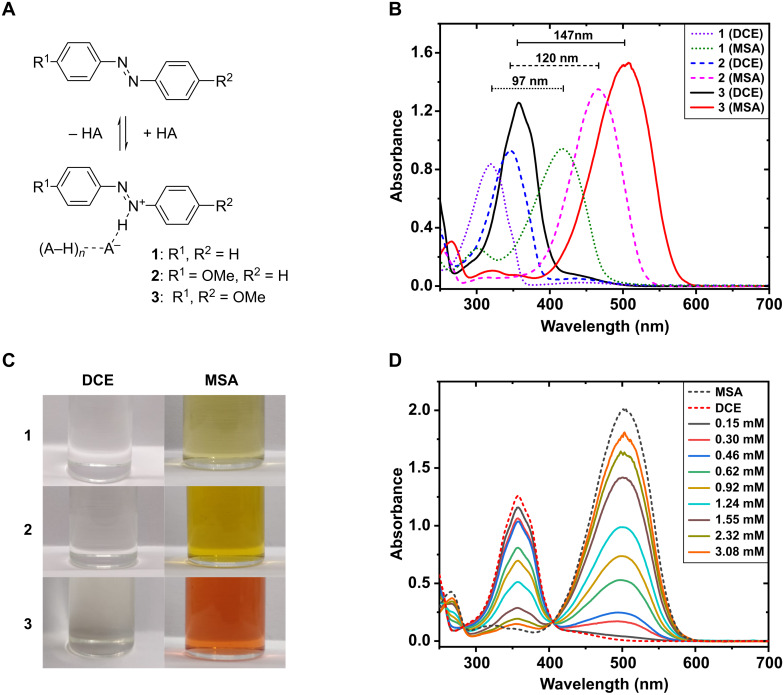
A) Protonation reaction scheme of azobenzene (**1**), 4-methoxyazobenzene (**2**), and 4,4'-dimethoxyazobenzene (**3**) with acid (HA). The parentheses indicate additional acid molecule(s) stabilizing the anion. B) Absorption spectra of **1**, **2**, and **3** in 1,2-dichloroethane (DCE) and methanesulfonic acid (MSA). C) Photographs showing the color change upon protonation, taken from 40 µM solutions of each azobenzene in DCE and MSA. D) The effect of MSA concentration on the degree of protonation of **3** in DCE at 25 °C. Dashed lines represent the spectra of 40 µM of **3** in pure DCE and MSA, calculated from the molar absorption coefficients. To account for slight azobenzene concentration variations the spectra have been dilution-corrected to 40 µM at 405 nm, which acts as a pseudo-isosbestic point between the neutral and protonated forms.

Due to the lone pairs on their nitrogen atoms, azobenzenes are weak bases. Their basicity can be increased by electron-donating substituents on the benzene rings. *para-*Alkoxy substitution effectively increases the electron density at the azo-nitrogens without introducing additional protonation sites. We opted for methoxy substitution on one or both benzene rings as the simplest alkoxy substituent. The protonation of azobenzene (**1**), 4-methoxyazobenzene (**2**), and 4,4'-dimethoxyazobenzene (**3**) is schematized in Figure 1A. In the presence of a strong acid, these compounds form an equilibrium of neutral and protonated species. Protonation leads to a notable red-shift in the absorption spectrum from 320–360 nm to 420–510 nm, as shown in Figure 1B using methanesulfonic acid (MSA) in 1,2-dichloroethane (DCE). We assume that in DCE alone, no protonation occurs. In MSA, on the other hand, full protonation is assumed. Stronger electron donation gives rise to larger red-shift, and hence more pronounced color changes, upon protonation, as illustrated in Figure 1C. Figure 1D illustrates the shift in protonation equilibrium with increasing acidity when MSA is added to DCE solution of **3** at 25 °C. Due to the strong acidity of MSA, moderate polarity of DCE, and the electron-donating substituents of **3**, only 76 equivalents of acid (3.08 mM) is needed for nearly full protonation as indicated by the almost complete disappearance of the absorption peak at 360 nm. Similar data for compounds **1** and **2** in MSA/DCE mixtures, as well as for **3** in MSA/acetonitrile mixture and for a weaker acid (trifluoroacetic acid), are shown in Supporting Information File 1, Figures S3–S6.

**Figure 2:**
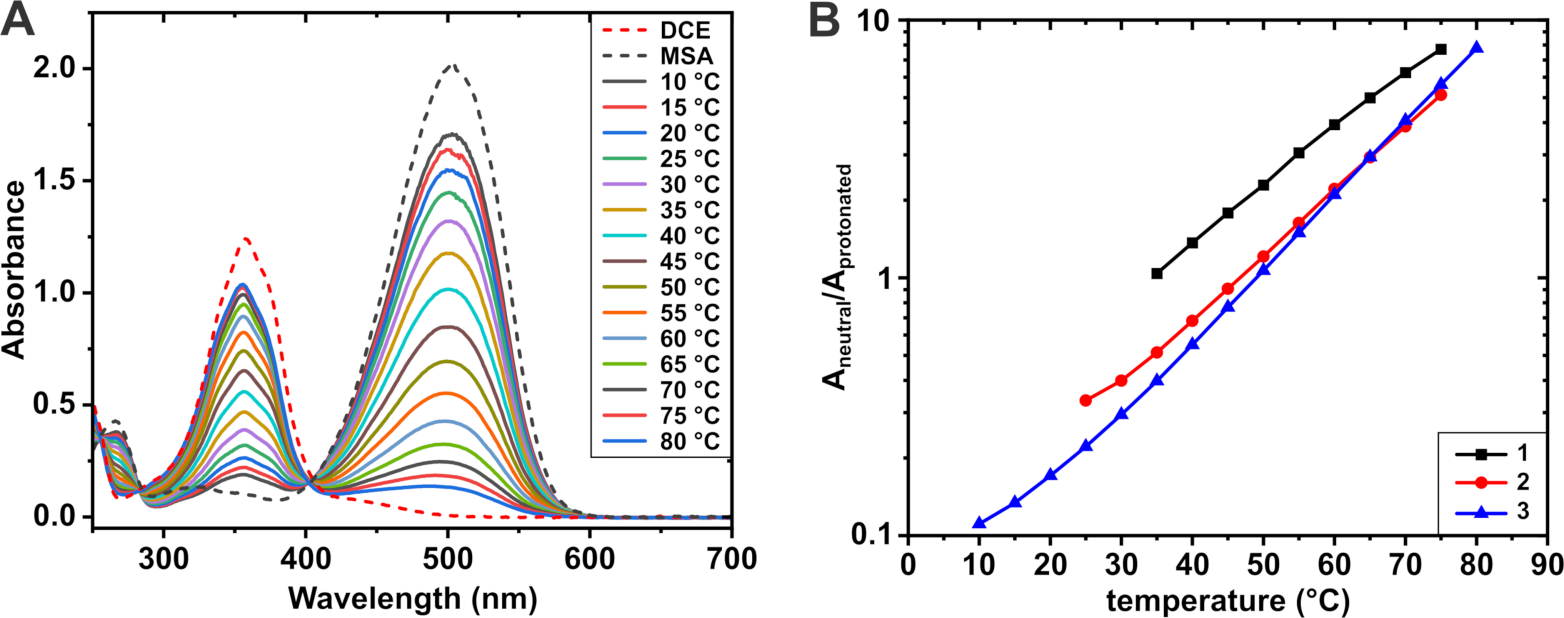
A) The effect of temperature on the degree of protonation of compound **3** (40 μM at 25 °C) in DCE with 1.44 mM MSA added. The reference spectra of **3** in pure DCE and pure MSA have been calculated for 40 µM solutions from molar absorption coefficients. B) Ratio of neutral and protonated π → π* absorption peak maxima for compounds **1**–**3** at different temperatures in MSA/DCE mixtures. Notice the logarithmic y-scale.

Given the large reversible absorption shift upon protonation, azobenzenes can act as pH indicators with tunable sensitivity through substituent modification. By combining a medium with temperature-dependent proton activity and an indicator molecule with systematic spectral changes, the system can be utilised for temperature sensing. Figure 2A shows the drastic change in the relative proportion of the neutral and protonated absorption peaks of **3** in DCE with 1.44 mM MSA over a 10–80 °C temperature range. Comparing the maximum absorbances at the neutral and protonated peaks provides a measure of sensitivity and optimal working range. Figure 2B presents this comparison for **1**–**3** in MSA/DCE solutions, calculated as the ratio between neutral and protonated peak maxima at each temperature (see Supporting Information File 1 for more details). Sensitivity is defined by the rate of change of ratio of absorption maxima (A_neutral_/A_protonated_) over a given temperature range, represented by the slope of the curves in Figure 2B. Within the optimal operation range, the rate of change should be both high and systematic. For example, in Figure 2B, the optimal range for **3** appears to be between 30 and 80 °C, where the rate of change is fastest and follows a clean, exponential trend. All three compounds show similar sensitivity in DCE, while the sensitivity of **3** is drastically reduced in MeCN (Figure S7 in Supporting Information File 1). This can be attributed to the less favorable protonation in MeCN as shown in Table 1.

**Table 1:** Computation ∆*G*° values for the complex and ion-pair formation of compounds **1**, **2**, and **3** with one or two molecules of methanesulfonic acid (MSA) in 1,2-dichloroethane (DCE) and acetonitrile (MeCN).

Complex or ion pair	∆*G*° in DCE (kcal/mol)	∆*G*° in MeCN (kcal/mol)

**1**-MSA	−0.16	0.31
**1**H^+^MSA^−^	1.17	1.04
**2**-MSA	−0.67	−0.30
**2**H^+^MSA^−^	−0.66	−1.03
**3**-MSA	−0.45	0.17
**3**H^+^MSA^−^	−1.59	−1.37
**1**-(MSA)_2_	1.62	2.45
**1**H^+^MSA^−^MSA	−2.55	−1.74
**2**H^+^MSA^−^MSA	−5.25	−4.53
**3**H^+^MSA^−^MSA	−5.81	−4.74

**Figure 3:**
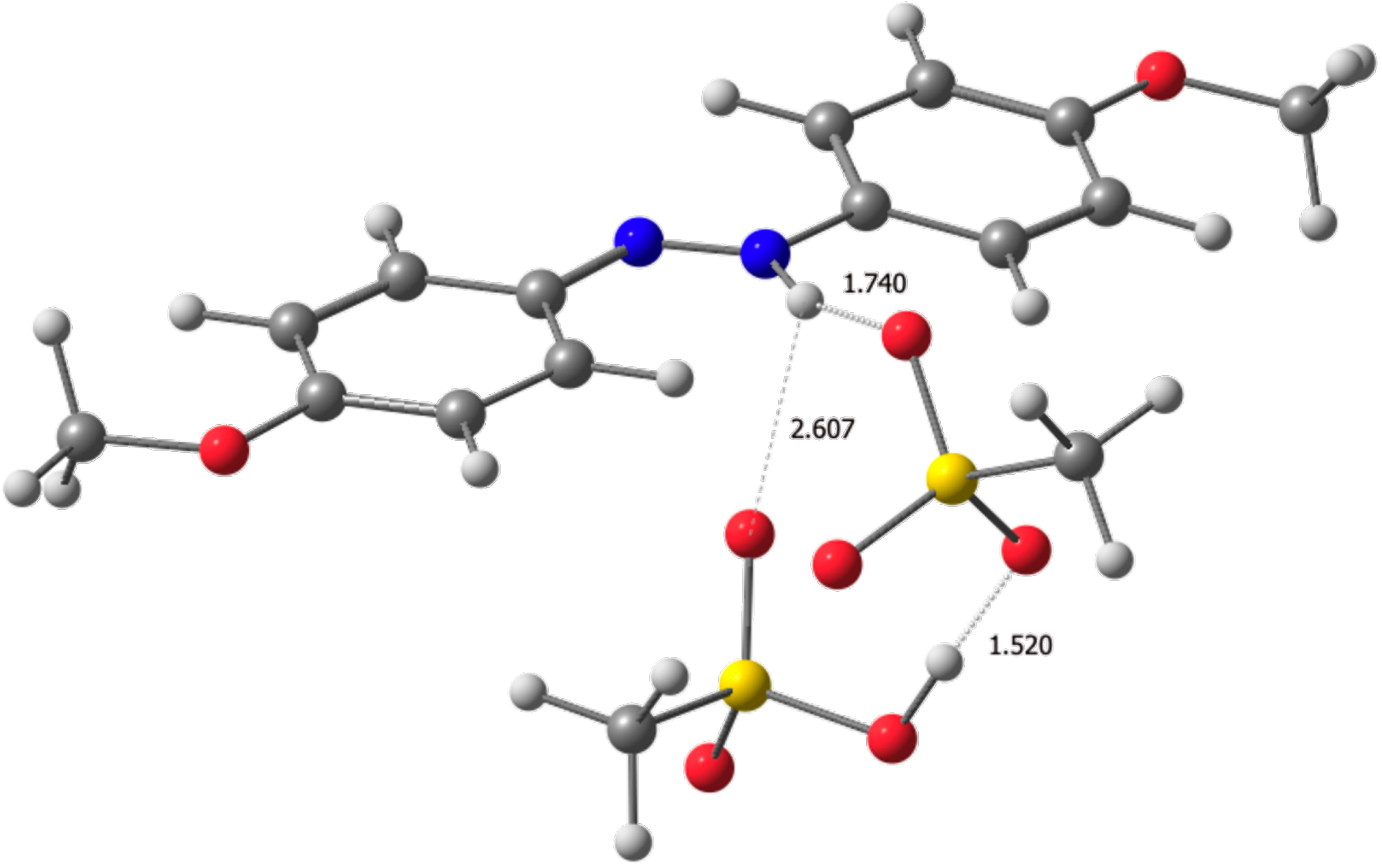
The geometry-optimized structure of **3**H^+^MSA^−^MSA.

The interaction between compounds **1**–**3** and MSA was studied computationally by geometry-optimizing their ion pairs, denoted as **1**H^+^MSA^−^, **2**H^+^MSA^−^, and **3**H^+^MSA^−^. Because the azo group contains two nitrogens with lone pairs and an excess of MSA was used in the experiments, geometry optimizations were also performed for complexes with two MSA molecules (**1**H^+^MSA^−^MSA, **2**H^+^MSA^−^MSA and **3**H^+^MSA^−^MSA). In addition to protonation, we also considered hydrogen-bonded complexes between **1**–**3** and MSA (**X**-MSA and **X**-(MSA)_2_, **X** = **1**, **2** or **3**). The geometry-optimized structure of **3**H^+^MSA^−^MSA is shown in Figure 3. In this structure, the second MSA molecule does not bind directly to the azo group but forms a hydrogen bond with the mesylate anion, and one of its oxygen atoms interacts with the **3**H^+^ ion. All geometry-optimized structures for compounds **1**–**3** are shown in Supporting Information File 1, Figures S19–S28).

Table 1 presents the calculated Gibbs free energy changes (∆*G*°) for the complexes. Among the **X**H^+^MSA^−^MSA (**X** = **1**, **2** or **3**) complexes, **3** shows the highest protonation propensity (∆*G*° = −5.81, −5.25, and −2.55 kcal/mol for **3**, **2**, and **1**, respectively). The protonation is more favorable in DCE (∆*G*° between −2.55 and −5.81 kcal/mol) than in MeCN (∆*G*° between −1.74 and −4.74 kcal/mol), highlighting solvent effects on reactivity. For **3** in DCE, the addition of a second MSA molecule substantially enhances protonation, lowering ∆*G*° from −1.59 to −5.81 kcal/mol.

Spectral changes upon protonation were also studied computationally using time-dependent density functional theory (TDDFT). Analysis of the molecular orbitals involved in the electronic transitions confirms that the absorption maxima for both neutral and protonated forms of compounds **1**–**3** correspond to π → π* transition (see Supporting Information File 1, Figures S9–S14). In accordance with the experimentally observed red-shift upon protonation (∆λ_max_ = 97–147 nm), the calculated spectra show comparable shifts (∆λ_max_ = 98–144 nm) between compounds **1**–**3** and their protonated counterparts (**1**H^+^–**3**H^+^; Figures S16–S18 in Supporting Information File 1).

The experimental results are enticing from the applications point of view but offer little insight into details of the azobenzene–acid interactions. DFT calculations were performed to study these interactions at the molecular level. Better understanding on the protonation process emerges from combining computational findings with experimental data, as discussed in the following section.

## Discussion

Upon protonation, azobenzene and the conjugate base of the acid form either an ion pair or dissociated ions. The ion pair can be a contact ion pair (CIP), where the ions remain in close proximity through hydrogen bonding or electrostatic interactions, or a solvent-separated ion pair (SIP), where solvent molecules intervene and weaken these interactions [30]. In a moderately polar aprotic solvent such as DCE, we assume the formation of CIP, as supported by computational results. This discussion focuses on compound **3**, which showed most promising characteristics for temperature sensing, including a larger red-shift upon protonation and a broader sensing range.

DFT calculations revealed that a second acid molecule significantly enhances protonation, prompting further investigation into the reaction stoichiometry. Upon protonation of **3**, the n → π* transition disappears and the π → π* transition red-shifts from 358 nm to 502 nm (Figure 1B), resulting in a visible color change from pale yellow to bright red. Since the protonated **3** shows minimal absorption between 300–400 nm (Figure 1B), the degree of protonation can be estimated by comparing the absorbance at 358 nm (neutral) and 502 nm (protonated). As the degree of protonation and initial concentration are known, the equilibrium constant (*K*) can be determined using Equation 1, provided that the reaction stoichiometry (*n*) is known.


[1] $$K=\frac{\left[3{\text{H}}^{+}{\text{MSA}}^{-}{\left(\text{MSA}\right)}_{n-1}\right]}{\left[3\right]{\left[\text{MSA}\right]}^{n}}\text{, }n\text{=0, 1, 2, }...$$

To determine the stoichiometry, the degree of protonation was measured for different acid concentrations and the corresponding *K* values were calculated. Using these *K* values, ∆*G*° can be determined using Equation 2. If the stoichiometry is correct, the calculated ∆*G*° values should not depend on the initial acid concentration.


[2] ΔG°=−RTlnK

We considered options where **3** forms ion pairs with one, two or three MSA molecules in DCE. The ∆*G*° values were calculated at three temperatures (15, 25, and 50 °C) for nine acid concentrations ([MSA]_0_ = 154–3080 µM, or 10–200 ppm; see Tables S1–S9 in Supporting Information File 1). For one or three acid molecules (*n* = 1 or 3), the ∆*G*° values varied with the initial acid concentration, indicating incorrect stoichiometry. However, for *n* = 2, constant ∆*G*° values were obtained, irrespective of initial acid concentration (Supporting Information File 1, Table S10). This suggests that upon protonation, **3** forms an ion pair with two MSA molecules (**3**H^+^MSA^−^MSA), as supported also by DFT calculations.

Assuming the 1:2 stoichiometry between **3** and MSA, the reaction thermodynamics was studied using van‘t Hoff equation, i.e., by plotting ln *K* versus 1/*T*. In an idealized case of temperature-independent enthalpy and entropy, this yields a straight line, allowing ∆*H*° and ∆*S*° to be extracted from the slope and intercept. However, a non-linear relationship was observed (see Supporting Information File 1, Figure S8), indicating temperature-dependent thermodynamics. To account for this, the polynomial form of the van 't Hoff equation (Equation 3) was used, showing that both ∆*H*° and ∆*S*° vary significantly with temperature (see Supporting Information File 1, Table S11).


[3] $$\begin{array}{l} \ln K=a+\frac{b}{T}+\frac{c}{T^{2}},\\ \Delta H{}^{\circ}=-R\cdot \left(b+\frac{2c}{T}\right)\text{, and }\\ \Delta S{}^{\circ}=R\cdot \left(a-\frac{c}{T^{2}}\right) \end{array}$$

The calculated ∆*H°* values ranged from −11.1 kcal/mol at 10 °C to −16.5 kcal/mol at 80 °C, while ∆*S°* decreased from −0.009 kcal/mol/K to −0.026 kcal/mol/K over the same range. These trends suggest that the ion pair is more strongly bound at higher temperatures. However, the increase in exothermicity is more than cancelled by the loss in entropy, resulting in a slight increase in ∆*G*° from −8.6 kcal/mol at 10 °C to −7.3 kcal/mol at 80 °C, in line with the observed temperature dependence of the protonation reaction.

The experimental and computational data indicate that the ions form a hydrogen-bonded ion pair (**3**H^+^MSA^−^MSA), which acts as a single thermodynamic unit. However, interactions with solvent molecules, underrepresented in implicit solvation models, may weaken this interaction in practice [31–32]. Since a solvent’s dielectric constant increases when temperature decreases, the higher ∆*H*° values at lower temperatures could reflect enhanced solvation, leading to weaker ion pairing. Thus, the hydrogen-bonded geometry predicted computationally may better represent conditions at higher temperatures, when the dielectric constant is lower. Future work could explore how additional substituents on the azobenzene core influence ion-pairing strength, offering a strategy to further control the protonation behavior and enhance the temperature-sensing performance.

With improved understanding of azobenzene–acid interactions and good agreement between computations and experiments, a design-driven approach to temperature sensing becomes increasingly feasible. The aim is to create systems with tailored absorption wavelengths and spectral shifts, sensitivity, and well-defined operational ranges. While the experimental results are promising, the use of a hazardous, chlorinated solvent is a major limitation, highlighting the need for safer, non-toxic alternatives. Computational design can play a key role in minimizing trial and error during system development and optimization. Looking even further, utilizing azobenzene protonation in solid matrices can open new opportunities for practical sensing applications.

## Experimental

1,2-Dichloroethane was purchased from Acros Organics (99.8% purity), acetonitrile from Honeywell (≥99.9% purity), methanesulfonic acid (MSA) from Sigma-Aldrich (≥99.5% purity), and trifluoroacetic acid (TFA) from TCI (≥99.0% purity). Azobenzene (98% purity) was bought from TCI and 4-methoxyazobenzene (≥98% purity) from Sigma-Aldrich. All of these were used as received. 4,4’-Dimethoxyazobenzene was synthesized according to the reaction depicted in Scheme S1 in Supporting Information File 1. The purity was confirmed by NMR spectroscopy (JNM-ECZR 500, JEOL).

UV–vis measurements were done with a Cary 60 UV–vis spectrometer (Agilent Technologies) in quartz cuvettes. Samples were prepared by first dissolving the azobenzene compounds in 1,2-dichloroethane to create stock solutions. MSA and TFA were added by creating acid–solvent mixtures of the desired acid concentration (e.g., 1000 ppm v/v in the target solvent). Samples of desired azobenzene concentration were then prepared by dilution from stock solutions into the desired acid–solvent mixture.

DFT calculations were performed with ORCA 6.0.1 [33] using the ωB97X-D4/def2-SVPD method. The conductor-like polarizable continuum model (CPCM) was used to account for the implicit solvation. Frequency calculations were performed for geometry-optimized structures to ensure that the structure was a true minimum energy structure (i.e., no imaginary frequencies). The molecular structures were visualized with ChemCraft version 1.8 (http://www.chemcraft.org) and Avogadro version 1.2.0 (http://www.avogadro.cc).

## Supporting Information

File 1 Reaction schemes and characterization, UV–vis absorption spectra, determination of the stoichiometry, determination of thermodynamic parameters for **3**H^+^MSA^−^MSA, molecular orbitals, calculated absorption spectra and geometry-optimized structures.

## Data Availability

All data that supports the findings of this study is available in the published article and/or the supporting information of this article.
