# Supplementary material for: Azobenzene protonation as a tool for temperature sensing
Source: Beilstein J Org Chem. 2025 Jul 28;21:1528–34. doi: 10.3762/bjoc.21.115 (PMC12320741; doi:10.3762/bjoc.21.115)
Supplement: File 1 — Reaction schemes and characterization, UV–vis absorption spectra, determination of the stoichiometry, determination of thermodynamic parameters for 3H+MSA−MSA, molecular orbitals, calculated absorption spectra and geometry-optimized structures. [file Beilstein_J_Org_Chem-21-1528-s001.pdf]

BEILSTEIN JOURNAL OF ORGANIC CHEMISTRY

Supporting Information

for

Azobenzene protonation as a tool for temperature sensing

Antti Siiskonen, Sami Vesamäki and Arri Priimagi

Beilstein J. Org. Chem. **2025,** *21,* 1528–1534. doi:10.3762/bjoc.21.115

Reaction schemes and characterization, UV–vis absorption spectra, determination of the stoichiometry, determination of thermodynamic parameters for $3H^+MSA^-MSA$, molecular orbitals, calculated absorption spectra and geometry-optimized structures

License and Terms: This is a supporting information file under the terms of the Creative Commons Attribution License (https://creativecommons.org/licenses/by/4.0). Please note that the reuse, redistribution and reproduction in particular requires that the author(s) and source are credited and that individual graphics may be subject to special legal provisions.
The license is subject to the *Beilstein Journal of Organic Chemistry* terms and conditions: (https://www.beilstein-journals.org/bjoc/terms)

Table of contents

1.Reaction schemes and characterisation S2

2.UV–vis absorption spectra S3

3.Determination of the stoichiometry of the protonation reaction S6

4.Determination of ΔH°, ΔS° and ΔG° for $3H^+MSA^-MSA$ formation S10

5.Molecular orbitals involved in the $\pi \rightarrow \pi^*$ transition S12

6.Calculated absorption spectra S13

7.Geometry-optimized structures S16

1. Reaction schemes and characterisation

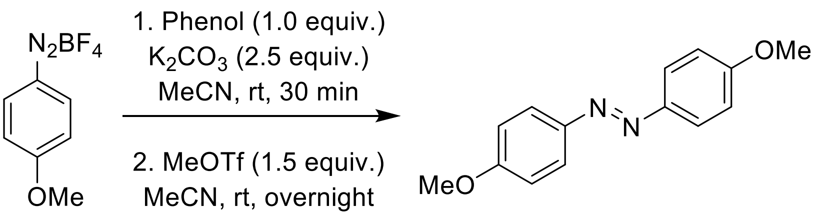

Scheme S1: The synthesis of 4,4'-dimethoxyazobenzene (**3**)

The synthetic work was carried out at the Tampere University. All reagents and solvents were commercial and purchased from Sigma Aldrich, TCI Europe or VWR. The NMR spectra were measured with a 500 MHz JEOL ECZR 500 spectrometer at 25 °C and processed with the JEOL Delta NMR software version 5.3.1 (Windows). The spectrum was referenced to the solvent peak (7.26 ppm).

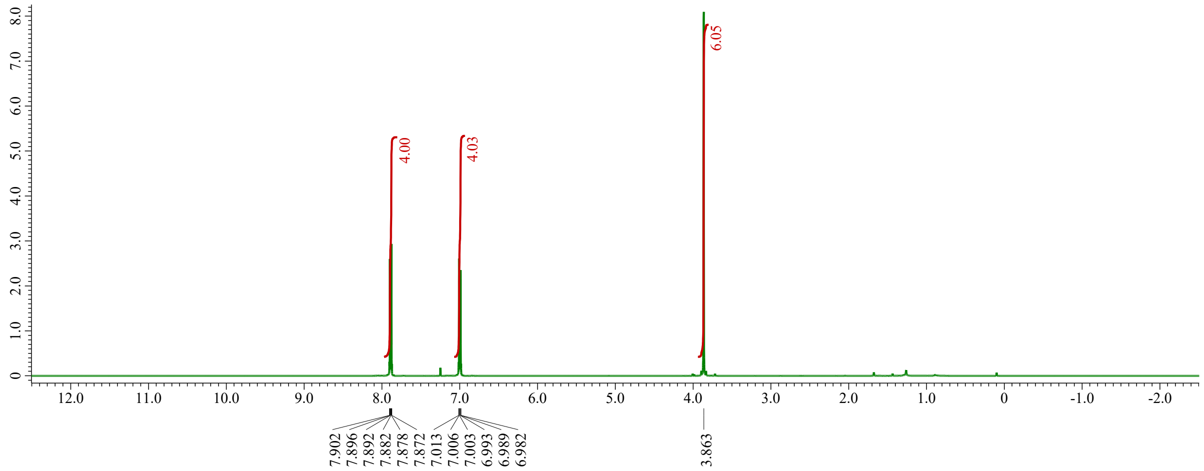

Figure S1: The ^{1}H NMR spectrum of the synthesised 4,4'-dimethoxyazobenzene (**3**).

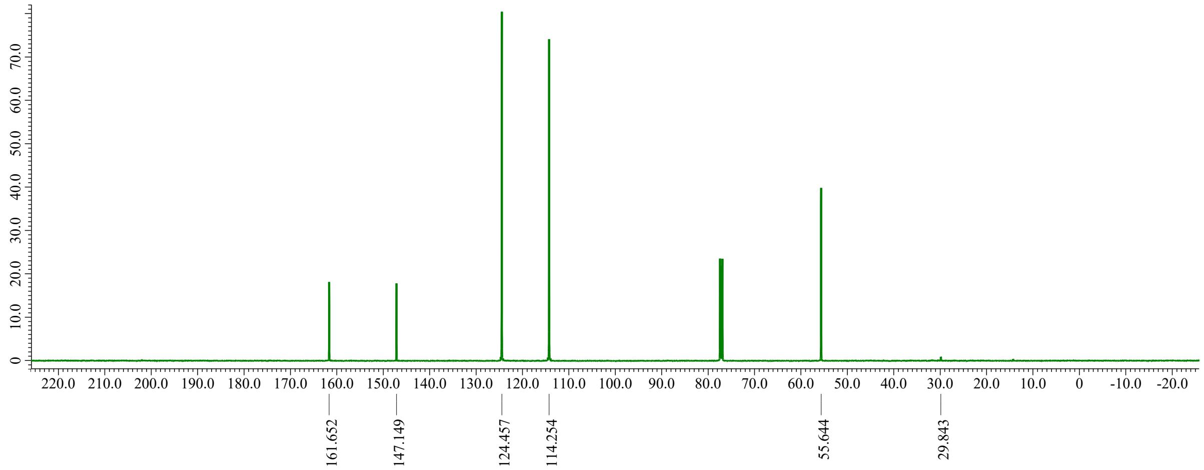

Figure S2: The ^{13}C NMR spectrum of the synthesised 4,4'-dimethoxyazobenzene (**3**).

2. UV–vis absorption spectra

Here are presented all the absorption spectra referenced but not shown in the main text. These include spectra for azobenzene (**1**) and 4-methoxyazobenzene (**2**) in 1,2-dichloroethane (DCE) with different amounts of methanesulfonic acid (MSA), spectra showing the effect of temperature on their absorption in MSA/DCE solutions, and the same spectra for 4,4'-dimethoxyazobenzene (**3**) in acetonitrile (MeCN) with MSA and trifluoroacetic acid (TFA).

Azobenzene (**1**)

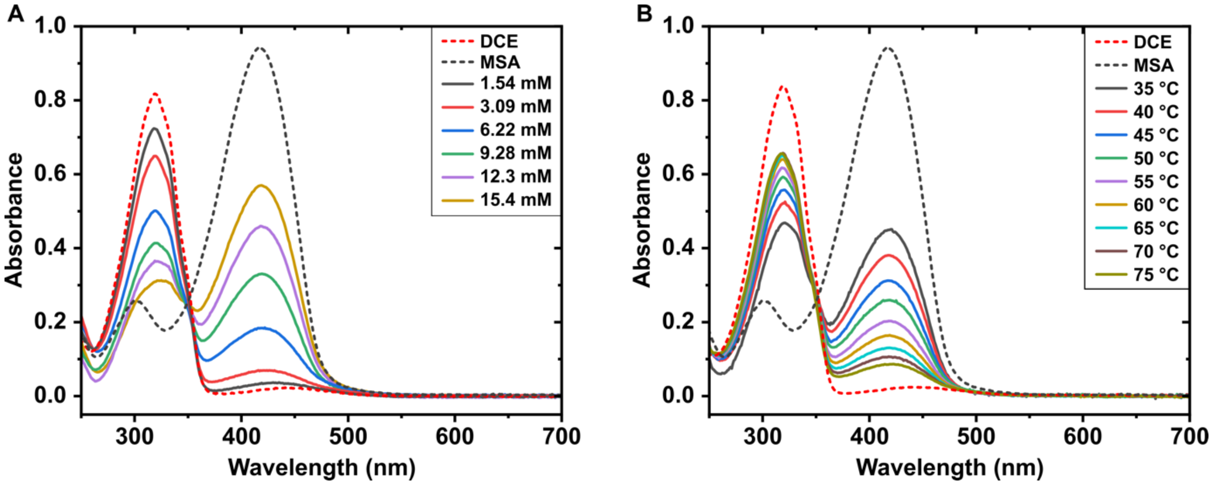

Figure S3: Absorption spectra of 40 µM azobenzene in DCE. A) The effect of the amount of MSA on the degree of protonation at 25 °C. B) The effect of temperature on the degree of protonation in a roughly 12 mM MSA solution.

The unsubstituted azobenzene **1** is a much weaker base than the methoxy substituted azobenzenes and thus higher amounts of MSA had to be used to shift the protonation equilibrium towards the protonated azobenzene. This presented a challenge with the chosen solvent and acid as MSA is not very soluble in DCE. MSA concentrations of over 1000 ppm (15.4 mM) separated at room temperature, and even 1000 ppm was not stable for extended periods of time. For the temperature series shown in Figure S3B, the target concentration of MSA was 15.4 mM, but comparison with the spectra in Figure S3A suggests that the actual MSA concentration is somewhat lower, closer to 12–13 mM. Also, the reason that the time series only goes down to 35 °C is that at low temperatures and high MSA concentrations the absorbance of undissolved MSA becomes dominant and even the slightest differences between the analysis sample and the baseline sample result in unusable spectra.

4-Methoxyazobenzene (**2**)

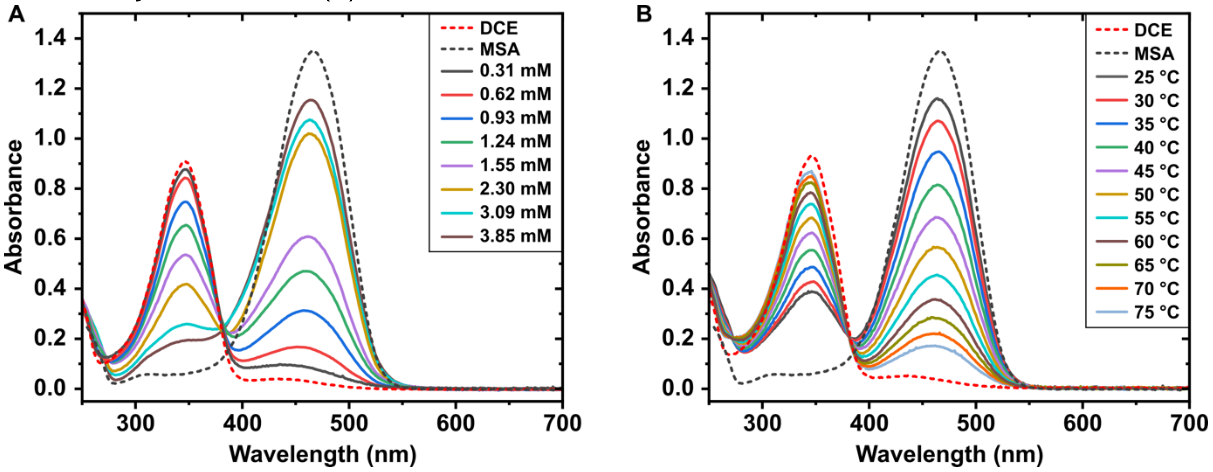

*Figure S4: Absorption spectra of 40 µM 4-methoxyazobenzene (**2**) in DCE. A) The effect of the amount of MSA on the degree of protonation at 25 °C. B) The effect of temperature on the degree of protonation in a roughly 2.5 mM MSA solution.*

In Figure S4A both peaks follow the expected trends when MSA concentration is increased. However, the 465 nm peak for a 2.3 mM solution is surprisingly high. This is likely due to errors in sample preparations. The absorbance of the mixture is sensitive to changes in azobenzene and MSA concentrations, and a slight variation in either can result in notable changes in the absorption spectrum. In this case, it is likely that the azobenzene and MSA concentrations were a bit higher than was aimed for, causing the 465 nm peak to be stronger both in relation to the 347 nm peak and the other samples.

For the temperature series a similar situation is true as for the unsubstituted azobenzene. The aimed MSA concentration was 3.09 mM but comparison with Figure S4A indicates actual MSA concentration somewhere between 2.3 mM and 3 mM.

4,4’-dimethoxyazobenzene (**3**)

The amount of acid needed for significant protonation in MeCN was significantly higher than in DCE, so the MSA amounts are presented as volume fractions (V-%). The temperature sensitivity is also much weaker in MeCN than in DCE.

<u>Acetonitrile and methanesulfonic acid</u>

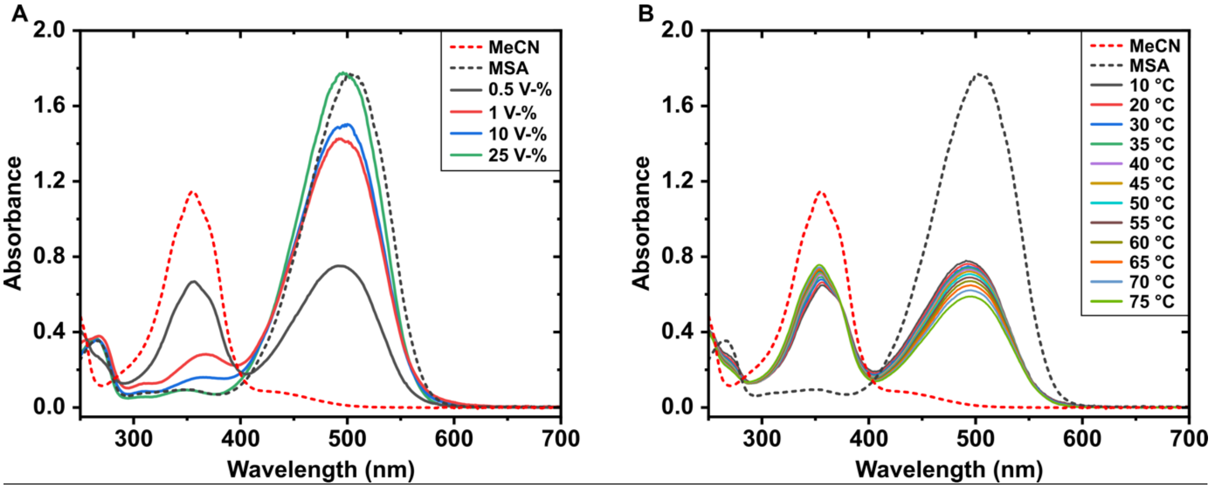

*Figure S5: Absorption spectra of 40 µM 4,4’-dimethoxyazobenzene (**3**) in MeCN. A) The effect of the amount of MSA on the degree of protonation at 25 °C. B) Effect of temperature on the degree of protonation in a 0.5 V-% MSA solution.*

<u>Acetonitrile and trifluoroacetic acid</u>

Using a weaker acid does not seem to affect the protonation other than more acid is needed to reach similar pH. With TFA the temperature sensitivity seems to be a bit stronger than with MSA.

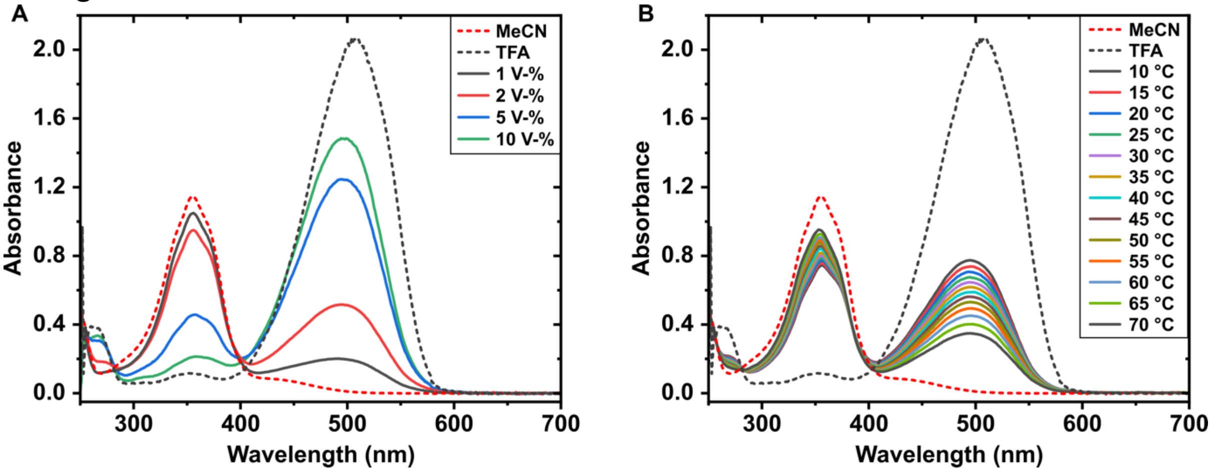

*Figure S6: Absorption spectra of 40 µM 4,4’-dimethoxyazobenzene (**3**) in MeCN. A) The effect of the amount of TFA on the degree of protonation at 25 °C. B) Effect of temperature on the degree of protonation in a roughly 3 V-% TFA solution.*

Proportional absorption

Same analysis as in main article's Figure 2B has been done to MeCN solution of 4,4'-dimethoxyazobenzene (**3**) to showcase temperature sensitivity. This is done by dividing neutral peak maximum with protonated peak maximum at each temperature. Results for all azobenzenes in MSA/DCE solutions have been included for ease of comparison.

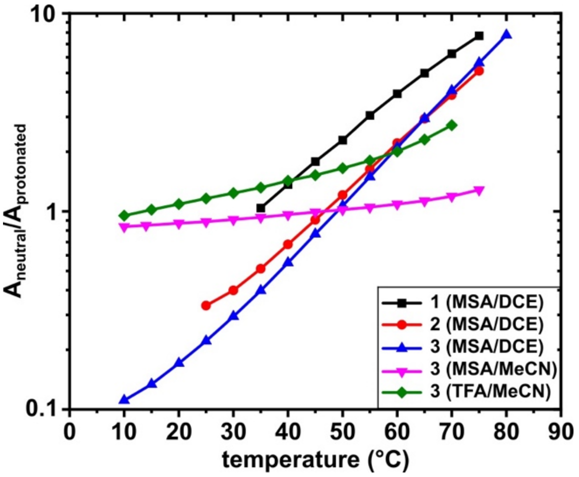

Figure S7: Proportion of the absorption peaks at different temperatures for different azobenzenes and acid/solvent mixtures. Calculated by dividing the shorter wavelength maximum with longer wavelength maximum. Notice logarithmic y-scale.

3. Determination of the stoichiometry of the protonation reaction

To determine whether more than one acid molecule is involved in the reaction, the $\Delta G°$ values were calculated for the reaction of 4,4'-dimethoxyazobenzene (**3**) with either 1, 2 or 3 MSA molecules in DCE. The reaction in the general form is presented in equation S1.

$$\mathbf{3} + n\,MSA \rightleftharpoons \mathbf{3}H^+MSA^-(MSA)_{n-1} \quad \text{(S1)}$$

The equilibrium constants for each reaction at three different temperatures (15, 25 and 50 °C) were calculated using the equation S2 and the $\Delta G°$ values were subsequently calculated using the equation S3.

$$K = \frac{[\mathbf{3}H^+MSA^-(MSA)_{n-1}]}{[\mathbf{3}][MSA]^n} \quad \text{(S2)}$$

$$\Delta G° = -RT\ln K \quad \text{(S3)}$$

1:1 stoichiometry:

$$\mathbf{3} + MSA \rightleftharpoons \mathbf{3}H^+MSA^-$$

Table S1: The measured protonation percentages with different amounts of MSA in DCE and the calculated equilibrium concentrations, lnK values and ΔG° values at 25 ℃ assuming 1:1 stoichiometry of 3 and MSA.

[**3**]$_0$ = 39.77 μM

MSA (ppm)	[MSA]$_0$ (μM)	Protonation (%)	[**3**H$^+$MSA$^-$] (μM)	[**3**] (μM)	[MSA] (μM)	ln*K*	Δ*G*° (kcal/mol)
10	153.1	1.65	0.66	39.11	152.45	4.700	-2.78
20	306.2	8.00	3.18	36.59	303.03	5.659	-3.35
30	459.3	11.84	4.71	35.06	454.61	5.689	-3.37
40	612.4	25.94	10.31	29.45	602.11	6.366	-3.77
60	918.6	36.38	14.47	25.30	904.17	6.450	-3.82
80	1224.9	48.93	19.46	20.31	1205.40	6.678	-3.96
100	1531.1	70.31	27.96	11.81	1503.11	7.363	-4.36
150	2296.6	80.81	32.14	7.63	2264.47	7.528	-4.46
200	3062.1	88.01	35.00	4.77	3027.14	7.793	-4.62

*Table S2: The measured protonation percentages with different amounts of MSA in DCE and the calculated equilibrium concentrations, lnK values and ΔG° values at 15 ℃ assuming 1:1 stoichiometry of **3** and MSA.*

[**3**]$_0$ = 40.23 μM

MSA (ppm)	[MSA]$_0$ (μM)	Protonation (%)	[**3**H$^+$MSA$^-$] (μM)	[**3**] (μM)	[MSA] (μM)	ln*K*	Δ*G*° (kcal/mol)
10	154.9	2.62	1.06	39.18	153.84	5.165	-3.06
20	309.8	13.51	5.43	34.80	304.35	6.241	-3.70
30	464.7	19.20	7.72	32.51	456.96	6.254	-3.71
40	619.6	38.25	15.39	24.84	604.18	6.933	-4.11
60	929.4	49.43	19.89	20.34	909.47	6.980	-4.14
80	1239.1	61.26	24.64	15.59	1214.50	7.172	-4.25
100	1548.9	78.82	31.71	8.52	1517.22	7.805	-4.62
150	2323.4	85.30	34.32	5.91	2289.08	7.838	-4.64
200	3097.9	92.97	37.41	2.83	3060.46	8.372	-4.96

*Table S3: The measured protonation percentages with different amounts of MSA in DCE and the calculated equilibrium concentrations, lnK values and ΔG° values at 50 ℃ assuming 1:1 stoichiometry of **3** and MSA.*

[**3**]$_0$ = 38.82 μM

MSA (ppm)	[MSA]$_0$ (μM)	Protonation (%)	[**3**H$^+$MSA$^-$] (μM)	[**3**] (μM)	[MSA] (μM)	ln*K*	Δ*G*° (kcal/mol)
10	149.5	0.47	0.18	38.64	149.29	3.449	-2.04
20	298.9	2.08	0.81	38.02	298.14	4.265	-2.53
30	448.4	2.70	1.05	37.78	447.37	4.128	-2.45
40	597.9	7.32	2.84	35.98	595.05	4.889	-2.90
60	896.8	11.43	4.44	34.39	892.40	4.974	-2.95
80	1195.8	18.91	7.34	31.48	1188.44	5.279	-3.13
100	1494.7	36.78	14.28	24.54	1480.44	5.974	-3.54
150	2242.1	50.96	19.79	19.04	2222.30	6.148	-3.64
200	2989.4	63.53	24.67	14.16	2964.78	6.376	-3.78

1:2 stoichiometry:

$$\mathbf{3} + 2\,MSA \rightleftharpoons \mathbf{3}H^{+}MSA^{-}MSA$$

*Table S4: The measured protonation percentages with different amounts of MSA in DCE and the calculated equilibrium concentrations, lnK values and ΔG° values at 25 ℃ assuming 1:2 stoichiometry of **3** and MSA.*

$[\mathbf{3}]_0$ = 39.77 μM

MSA (ppm)	$[MSA]_0$ (μM)	Protonation (%)	$[\mathbf{3}H^+MSA^-MSA]$ (μM)	[**3**] (μM)	[MSA] (μM)	ln$K$	$\Delta G°$ (kcal/mol)
10	153.1	1.65	0.66	39.11	151.80	13.498	-8.00
20	306.2	8.00	3.18	36.59	299.85	13.782	-8.17
30	459.3	11.84	4.71	35.06	449.90	13.406	-7.94
40	612.4	25.94	10.31	29.45	591.80	13.815	-8.19
60	918.6	36.38	14.47	25.30	889.70	13.490	-7.99
80	1224.9	48.93	19.46	20.31	1185.94	13.431	-7.96
100	1531.1	70.31	27.96	11.81	1475.14	13.900	-8.24
150	2296.6	80.81	32.14	7.63	2232.33	13.647	-8.09
200	3062.1	88.01	35.00	4.77	2992.14	13.617	-8.07

*Table S5: The measured protonation percentages with different amounts of MSA in DCE and the calculated equilibrium concentrations, lnK values and ΔG° values at 15 ℃ assuming 1:2 stoichiometry of **3** and MSA.*

$[\mathbf{3}]_0$ = 40.23 μM

MSA (ppm)	$[MSA]_0$ (μM)	Protonation (%)	$[\mathbf{3}H^+MSA^-MSA]$ (μM)	[**3**] (μM)	[MSA] (μM)	ln$K$	$\Delta G°$ (kcal/mol)
10	154.9	2.62	1.06	39.18	152.78	13.959	-8.27
20	309.8	13.51	5.43	34.80	298.92	14.374	-8.52
30	464.7	19.20	7.72	32.51	449.23	13.979	-8.28
40	619.6	38.25	15.39	24.84	588.80	14.396	-8.53
60	929.4	49.43	19.89	20.34	889.59	14.027	-8.31
80	1239.1	61.26	24.64	15.59	1189.86	13.926	-8.25
100	1548.9	78.82	31.71	8.52	1485.51	14.338	-8.50
150	2323.4	85.30	34.32	5.91	2254.76	13.948	-8.26
200	3097.9	92.97	37.41	2.83	3023.05	14.186	-8.40

*Table S6: The measured protonation percentages with different amounts of MSA in DCE and the calculated equilibrium concentrations, lnK values and ΔG° values at 50 ℃ assuming 1:2 stoichiometry of **3** and MSA.*

$[\mathbf{3}]_0$ = 38.82 μM

MSA (ppm)	$[MSA]_0$ (μM)	Protonation (%)	$[\mathbf{3}H^+MSA^-MSA]$ (μM)	[**3**] (μM)	[MSA] (μM)	ln$K$	$\Delta G°$ (kcal/mol)
10	149.5	0.47	0.18	38.64	149.11	12.261	-7.26
20	298.9	2.08	0.81	38.02	297.33	12.389	-7.34
30	448.4	2.70	1.05	37.78	446.32	11.845	-7.02
40	597.9	7.32	2.84	35.98	592.20	12.325	-7.30
60	896.8	11.43	4.44	34.39	887.96	12.005	-7.11

80	1195.8	18.91	7.34	31.48	1181.10	12.027	-7.13
100	1494.7	36.78	14.28	24.54	1466.17	12.509	-7.41
150	2242.1	50.96	19.79	19.04	2202.52	12.275	-7.27
200	2989.4	63.53	24.67	14.16	2940.12	12.214	-7.24

1:3 stoichiometry:

$$\mathbf{3} + 3\,MSA \rightleftharpoons \mathbf{3}H^{+}MSA^{-}MSA_2$$

*Table S7: The measured protonation percentages with different amounts of MSA in DCE and the calculated equilibrium concentrations, lnK values and ΔG° values at 25 ℃ assuming 1:3 stoichiometry of **3** and MSA.*

$[\mathbf{3}]_0$ = 39.77 μM

MSA (ppm)	$[MSA]_0$ (μM)	Protonation (%)	$[\mathbf{3}H^+MSA^-MSA]$ (μM)	[**3**] (μM)	[MSA] (μM)	ln*K*	Δ*G*° (kcal/mol)
10	153.1	1.65	0.66	39.11	151.14	22.304	-13.21
20	306.2	8.00	3.18	36.59	296.67	21.926	-12.99
30	459.3	11.84	4.71	35.06	445.19	21.144	-12.53
40	612.4	25.94	10.31	29.45	581.48	21.301	-12.62
60	918.6	36.38	14.47	25.30	875.24	20.564	-12.18
80	1224.9	48.93	19.46	20.31	1166.48	20.218	-11.98
100	1531.1	70.31	27.96	11.81	1447.18	20.477	-12.13
150	2296.6	80.81	32.14	7.63	2200.19	19.795	-11.73
200	3062.1	88.01	35.00	4.77	2957.14	19.464	-11.53

*Table S8: The measured protonation percentages with different amounts of MSA in DCE and the calculated equilibrium concentrations, lnK values and ΔG° values at 15 ℃ assuming 1:3 stoichiometry of **3** and MSA.*

$[\mathbf{3}]_0$ = 40.23 μM

MSA (ppm)	$[MSA]_0$ (μM)	Protonation (%)	$[\mathbf{3}H^+MSA^-MSA]$ (μM)	[**3**] (μM)	[MSA] (μM)	ln*K*	Δ*G*° (kcal/mol)
10	154.9	2.62	1.06	39.18	151.73	22.766	-13.49
20	309.8	13.51	5.43	34.80	293.48	22.544	-13.36
30	464.7	19.20	7.72	32.51	441.51	21.739	-12.88
40	619.6	38.25	15.39	24.84	573.41	21.913	-12.98
60	929.4	49.43	19.89	20.34	869.70	21.119	-12.51
80	1239.1	61.26	24.64	15.59	1165.21	20.723	-12.28
100	1548.9	78.82	31.71	8.52	1453.80	20.915	-12.39
150	2323.4	85.30	34.32	5.91	2220.44	20.089	-11.90
200	3097.9	92.97	37.41	2.83	2985.65	20.024	-11.86

*Table S9: The measured protonation percentages with different amounts of MSA in DCE and the calculated equilibrium concentrations, lnK values and ΔG° values at 50 ℃ assuming 1:3 stoichiometry of **3** and MSA.*

$[\mathbf{3}]_0$ = 38.82 μM

MSA (ppm)	$[MSA]_0$ (μM)	Protonation (%)	[**3**H^+MSA^-MSA] (μM)	[**3**] (μM)	[MSA] (μM)	ln*K*	Δ*G*° (kcal/mol)
10	149.5	0.47	0.18	38.64	148.93	21.075	-12.49
20	298.9	2.08	0.81	38.02	296.52	20.517	-12.16
30	448.4	2.70	1.05	37.78	445.27	19.566	-11.59
40	597.9	7.32	2.84	35.98	589.36	19.772	-11.71
60	896.8	11.43	4.44	34.39	883.52	19.047	-11.29
80	1195.8	18.91	7.34	31.48	1173.76	18.787	-11.13
100	1494.7	36.78	14.28	24.54	1451.89	19.063	-11.29
150	2242.1	50.96	19.79	19.04	2182.73	18.420	-10.91
200	2989.4	63.53	24.67	14.16	2915.45	18.068	-10.71

*Table S10: The experimental ΔG° values, average values and standard deviations (SD) with **3**:MSA stoichiometries of 1:1, 1:2 and 1:3 at 15, 25 and 50 ℃.*

	Δ*G*° (kcal/mol)								
	1:1 **3**:MSA			1:2 **3**:MSA			1:3 **3**:MSA		
MSA (ppm)	15 °C	25 °C	50 °C	15 °C	25 °C	50 °C	15 °C	25 °C	50 °C
10	-3.06	-2.78	-2.04	-8.27	-8.00	-7.26	-13.49	-13.21	-12.49
20	-3.70	-3.35	-2.53	-8.52	-8.17	-7.34	-13.36	-12.99	-12.16
30	-3.71	-3.37	-2.45	-8.28	-7.94	-7.02	-12.88	-12.53	-11.59
40	-4.11	-3.77	-2.90	-8.53	-8.19	-7.30	-12.98	-12.62	-11.71
60	-4.14	-3.82	-2.95	-8.31	-7.99	-7.11	-12.51	-12.18	-11.29
80	-4.25	-3.96	-3.13	-8.25	-7.96	-7.13	-12.28	-11.98	-11.13
100	-4.62	-4.36	-3.54	-8.50	-8.24	-7.41	-12.39	-12.13	-11.29
150	-4.64	-4.46	-3.64	-8.26	-8.09	-7.27	-11.90	-11.73	-10.91
200	-4.96	-4.62	-3.78	-8.40	-8.07	-7.24	-11.86	-11.53	-10.71
average	-4.13	-3.83	-2.99	**-8.37**	**-8.07**	**-7.23**	-12.63	-12.32	-11.48
SD	0.58	0.60	0.59	**0.12**	**0.11**	**0.12**	0.59	0.56	0.58

4. Determination of Δ*H*°, Δ*S*° and Δ*G*° for $3H^+MSA^-MSA$ formation

To study the thermodynamics of the reaction of **3** with 2 MSA molecules, the degree of protonation was measured at 15 temperatures in the range 10–80°C. The *K* values were then calculated according to equation 2. The ln*K* values were then plotted as a function of the inverse of temperature (1/T) (Figure S8) and the parameters a, b and c in equation S4 were obtained from the polynomial fit. The Δ*H*° and Δ*S*° values were calculated using the equation S4 and the Δ*G*° values were calculated using the equation S3. The calculated ln*K*, Δ*H*°, Δ*S*° and Δ*G*° values are shown in table S11.

$$\ln K = a + \frac{b}{T} + \frac{c}{T^2},\ \Delta H° = -R \cdot \left(b + \frac{2c}{T}\right) \text{ and } \Delta S° = R \cdot \left(a - \frac{c}{T^2}\right) \quad \text{(S4)}$$

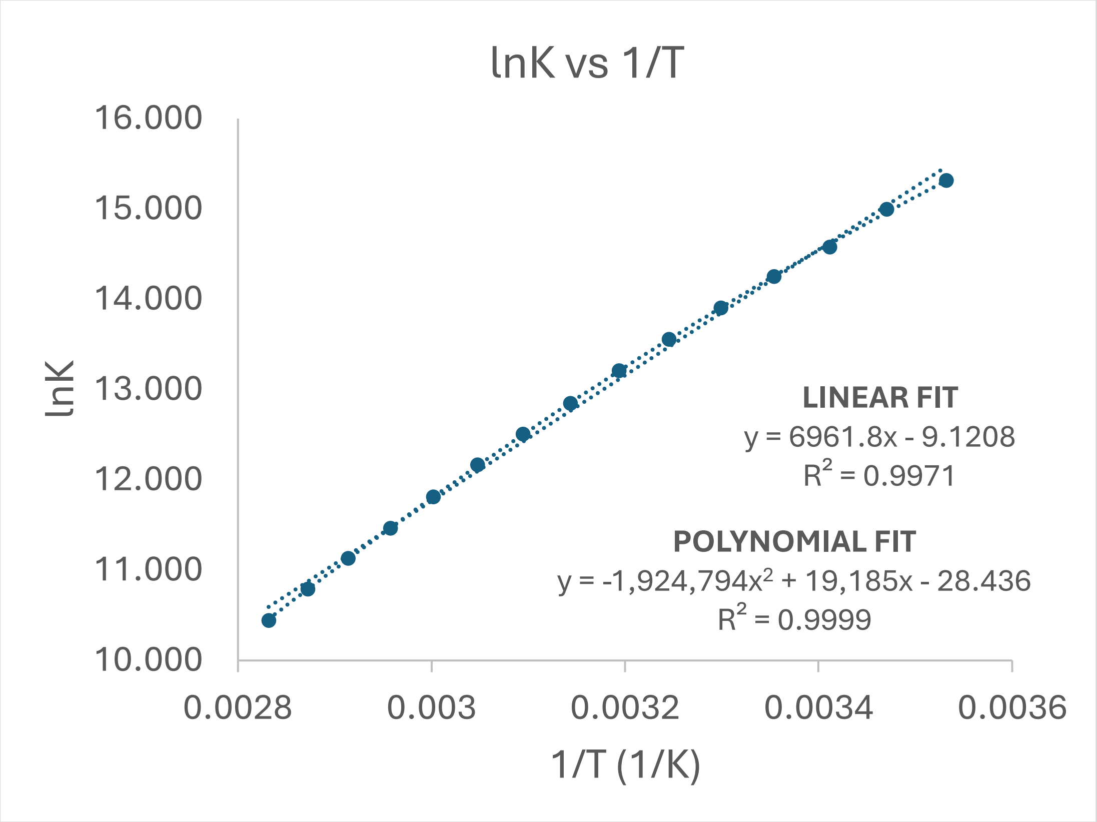

Figure S8: The van 't Hoff plot for the 3H+MSA-MSA formation showing the linear and polynomial fits.

Table S11: The experimental lnK, ΔH°, ΔS° and ΔG° values for the formation of ***3****H*$^{+}$*MSA*$^{-}$*MSA in the temperature range 283.15 - 353.15 K (10 – 80 ℃)*

T_c (°C)	T (K)	ln*K*	Δ*H*° (kcal/mol)	Δ*S*° (kca/mol)	Δ*G*° (kcal/mol)
10	283.15	15.312	-11.11	-0.0088	-8.62
15	288.15	14.992	-1158	-0.0104	-8.57
20	293.15	14.578	-12.03	-0.0120	-8.51
25	298.15	14.249	-12.47	-0.0135	-8.45
30	303.15	13.902	-12.89	-0.0149	-8.38
35	308.15	13.558	-13.30	-0.0162	-8.30
40	313.15	13.204	-13.70	-0.0175	-8.21
45	318.15	12.846	-14.08	-0.0187	-8.12
50	323.15	12.504	-14.45	-0.0199	-8.03
55	328.15	12.162	-14.81	-0.0210	-7.93
60	333.15	11.811	-15.16	-0.0220	-7.82
65	338.15	11.463	-15.50	-0.0231	-7.70
70	343.15	11.130	-15.83	-0.0240	-7.59
75	348.15	10.793	-16.15	-0.0250	-7.46
80	353.15	10.446	-16.46	-0.0258	-7.34

5. Molecular orbitals involved in the $\pi \rightarrow \pi^*$ transition

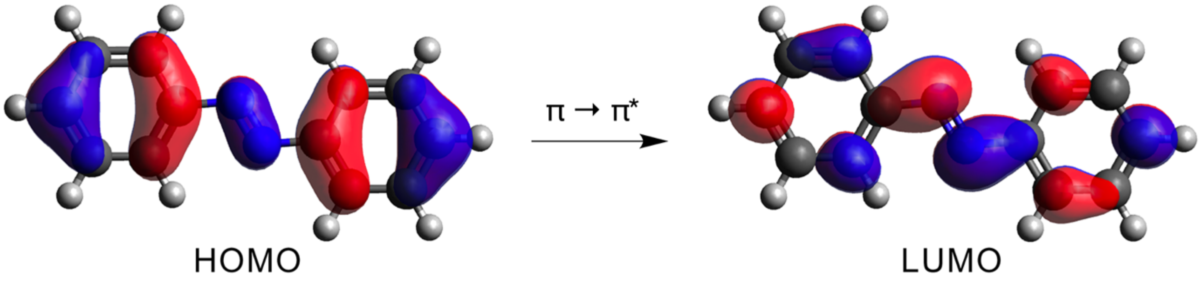

*Figure S9: The molecular orbitals of **1** involved in the $\pi \rightarrow \pi^*$ transition.*

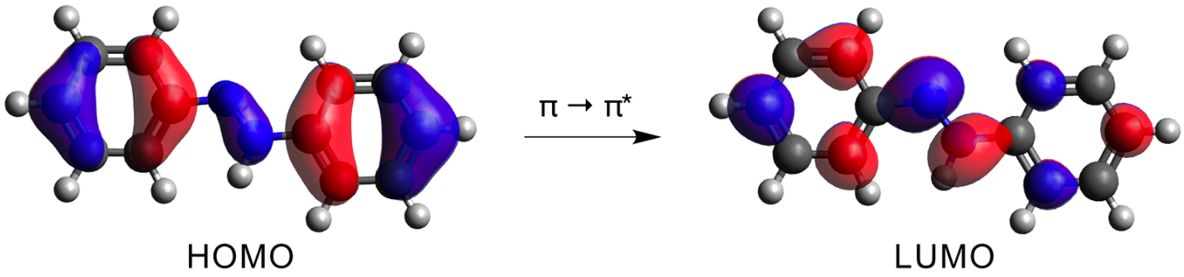

*Figure S10: The molecular orbitals of **1**H^+ involved in the $\pi \rightarrow \pi^*$ transition.*

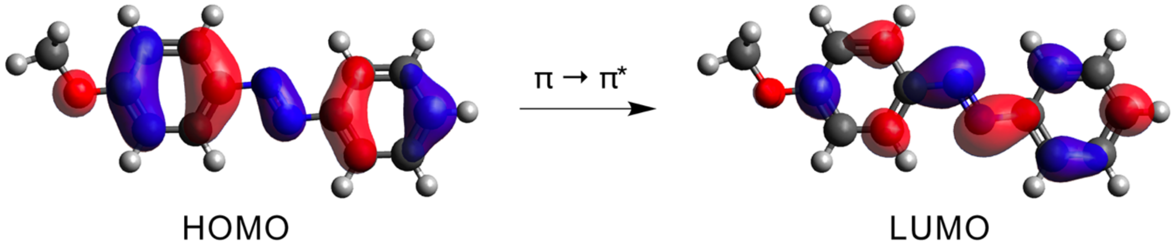

*Figure S11: The molecular orbitals of **2** involved in the $\pi \rightarrow \pi^*$ transition.*

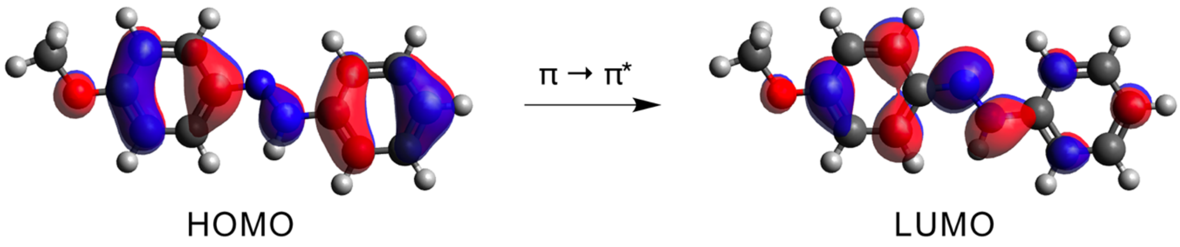

*Figure S12: The molecular orbitals of **2**H^+ involved in the $\pi \rightarrow \pi^*$ transition.*

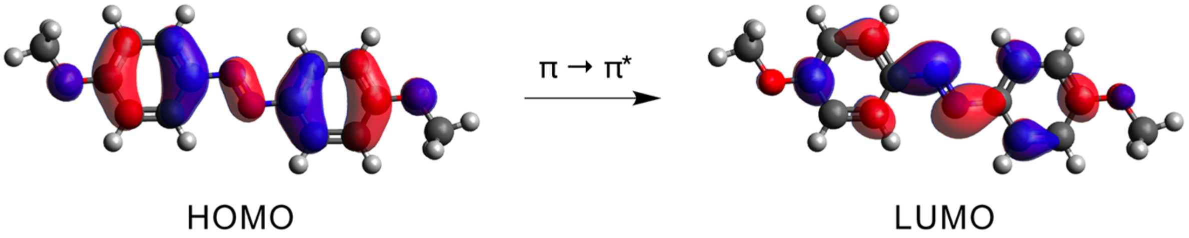

*Figure S13: The molecular orbitals of **3** involved in the $\pi \rightarrow \pi^*$ transition.*

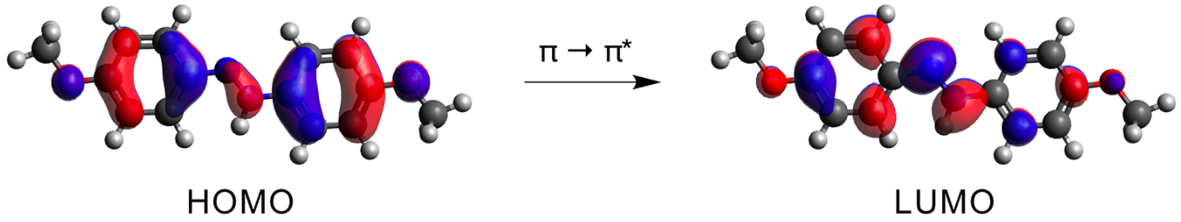

*Figure S14: The molecular orbitals **3**H$^+$ involved in the $\pi \rightarrow \pi^*$ transition.*

6. Calculated absorption spectra

The absorption spectra of neutral and protonated **1**, **2**, and **3** were calculated using the Time-Dependent Density Functional Theory (TDDFT) and the method SCS-RSX-QIDH/def2-SVPD, employing the Tamm-Dancoff approximation (TDA) which is the default option for TDDFT calculations in ORCA 6. The lowest 8 singlet excited states were considered (nroots = 8). The calculations were performed in DCE using the CPCM solvation model. The calculated absorption maxima were systematically blueshifted compared to the experimental values. However, a good linear fit was obtained when plotting the experimental values as a function of the calculated values (Figure S15). The experimental and calculated absorption maxima of the $\pi \rightarrow \pi^*$ transition are shown in Table S12 together with the calculated absorption maxima after fitting.

The program *orca_mapspc* included in the ORCA 6 package was used to turn the spectra calculated using TDDFT into plottable format. The full-width at half-maximum value (FWHM) of 3000 was used to widen the peaks. The calculated absorption spectra for the neutral and protonated **1**, **2** and **3** are shown in Figures S16–S18.

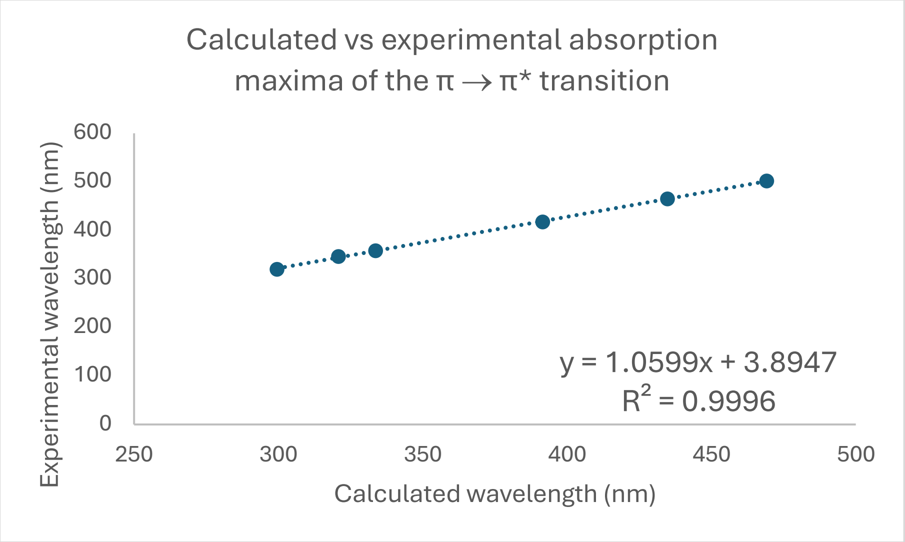

*Figure S15: The experimental absorption maxima of the $\pi \rightarrow \pi^*$ transition as a function of the calculated maxima for neutral and protonated **1**, **2**, and **3**.*

*Table S12: The experimental and calculated (with and without fitting) absorption maxima of the $\pi \rightarrow \pi^*$ transition for neutral and protonated **1**, **2** and **3**.*

Compound	experimental $\pi \rightarrow \pi^*$	calculated $\pi \rightarrow \pi^*$	calculated $\pi \rightarrow \pi^*$ after fitting
**1**	320	299.5	321.3
**1**H^+	417	391.5	418.8
**2**	346	320.8	343.9
**2**H^+	464	434.6	464.5
**3**	358	333.6	357.5
**3**H^+	502	469.0	501.0

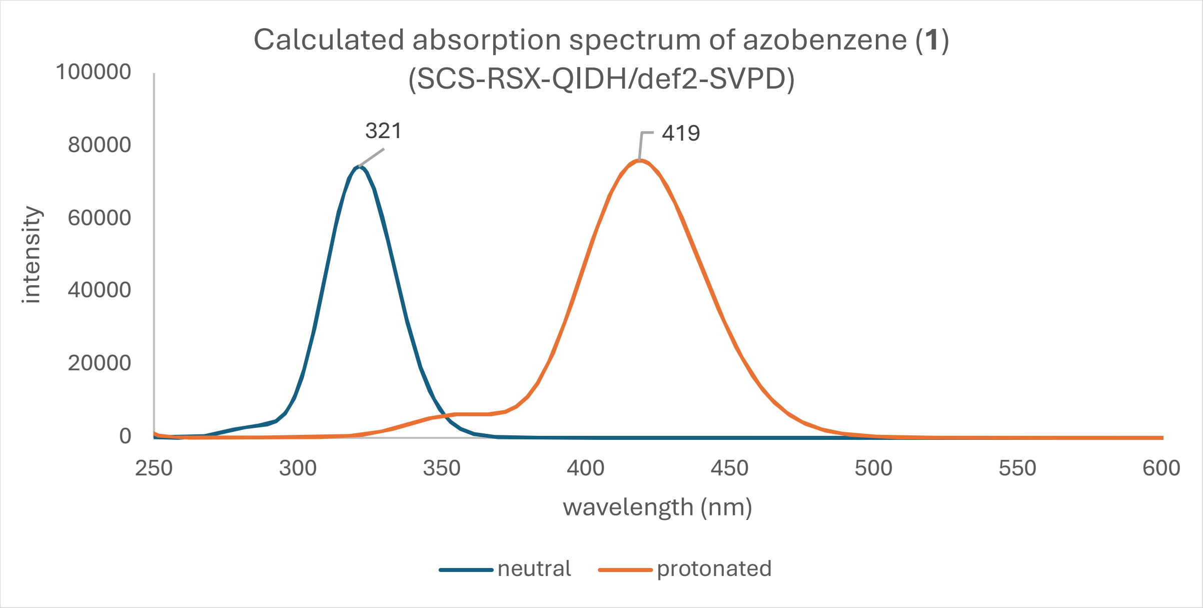

*Figure S16: The calculated absorption spectrum of neutral and protonated **1** in DCE (SCS-RSX-QIDH/def2-SVPD).*

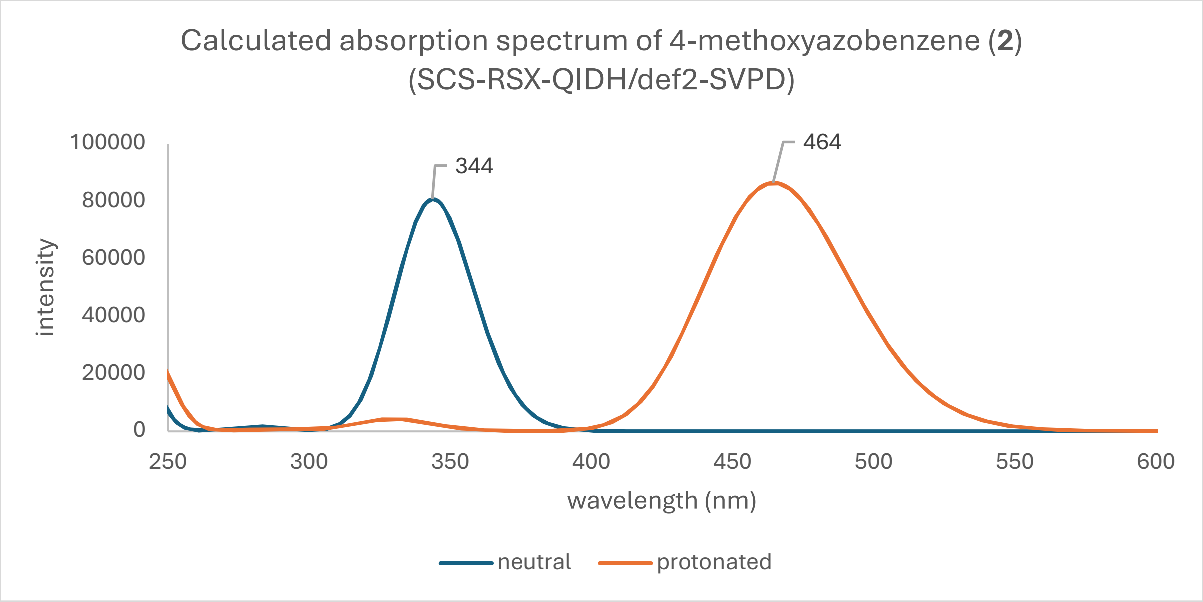

*Figure S17: The calculated absorption spectrum of neutral and protonated **2** in DCE (SCS-RSX-QIDH/def2-SVPD).*

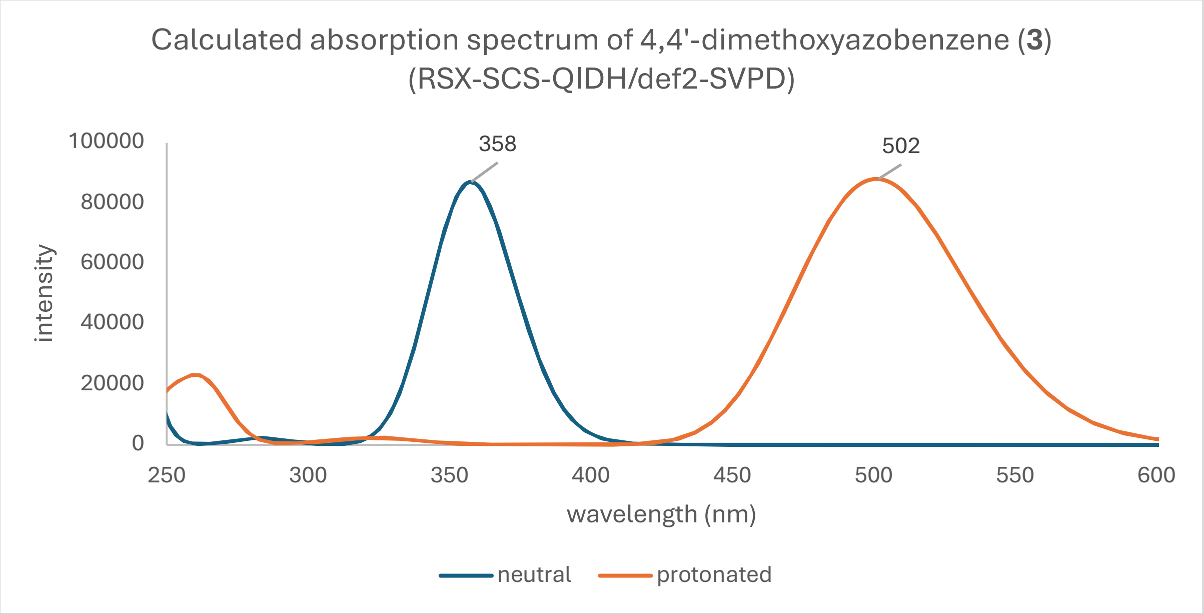

*Figure S18: The calculated absorption spectrum of neutral and protonated **3** in DCE (SCS-RSX-QIDH/def2-SVPD).*

7. Geometry-optimized structures

The geometry-optimized structures were obtained in two steps. In the first step, the DOCKER program in ORCA 6 was used to find reasonable conformations for the complexes. The GFN2-xTB method was used for that step. In the second step, the obtained conformations were geometry-optimized using the DFT method ωB97X-D4/def2-SVPD. The lowest energy conformations of the geometry-optimized structures are shown from two angles in Figures S19–S28. The distances between selected atoms are shown in Ångströms.

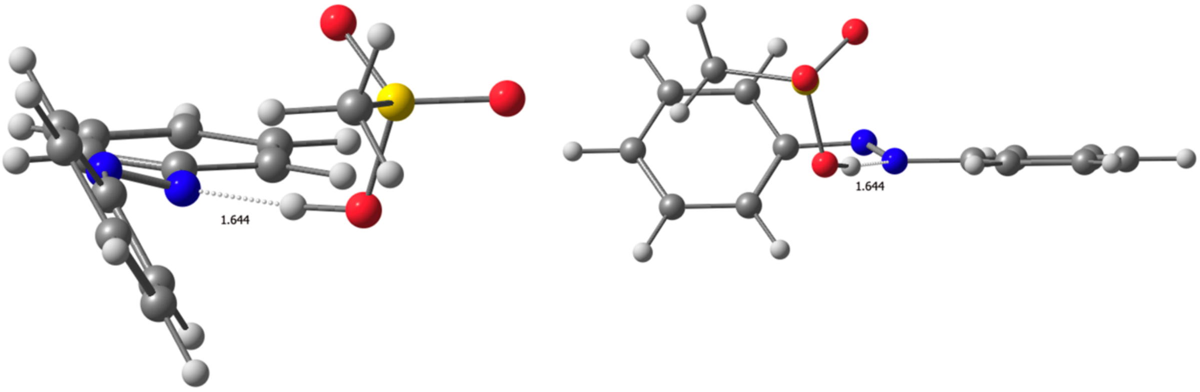

*Figure S19: Geometry-optimized structure of **1**-MSA.*

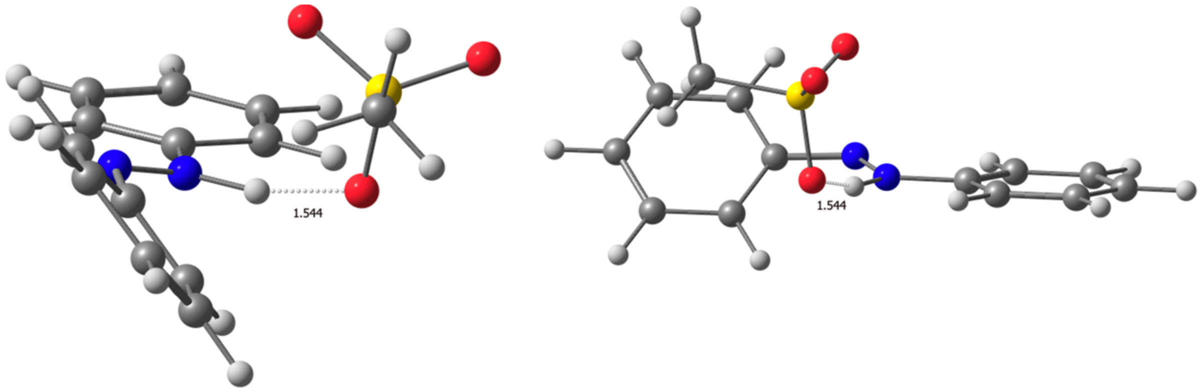

Figure S20: Geometry-optimized structure of ***1****H⁺MSA⁻.*

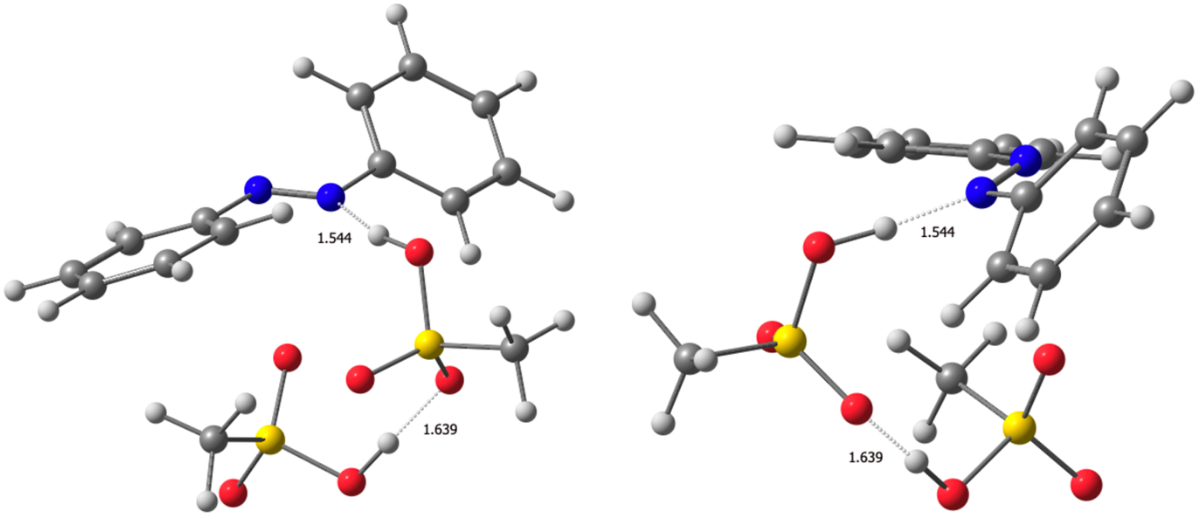

Figure S21: Geometry-optimized structure of ***1****-(MSA)$_2$.*

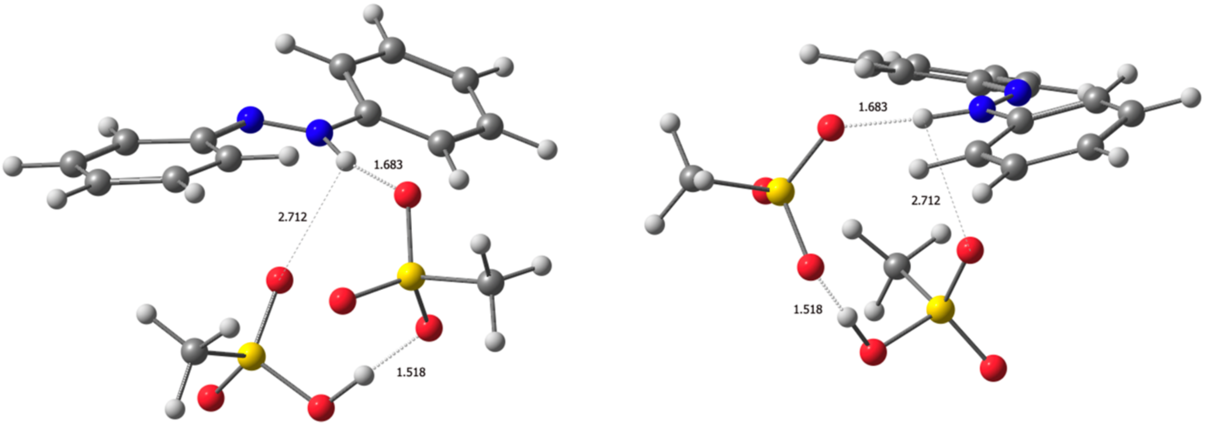

Figure S22: Geometry-optimized structure of ***1****H⁺MSA⁻MSA.*

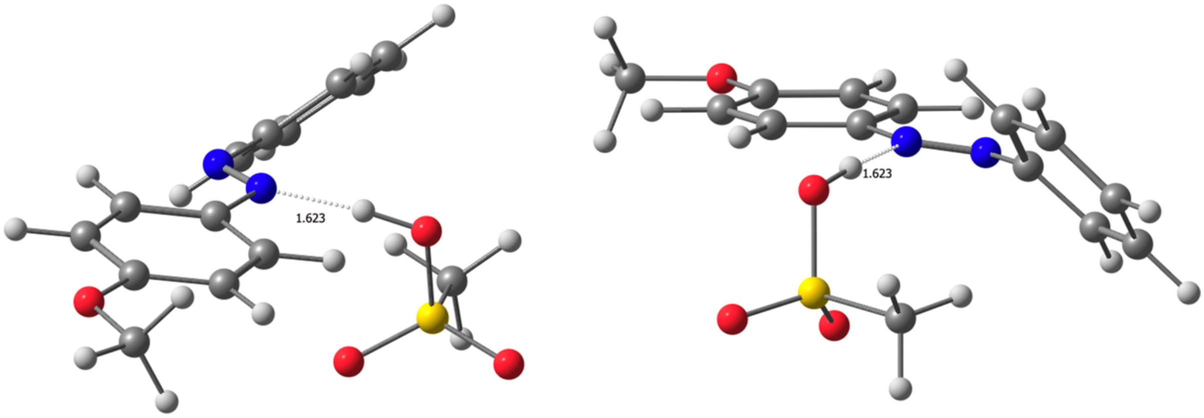

Figure S23: Geometry-optimized structure of ***2****-MSA.*

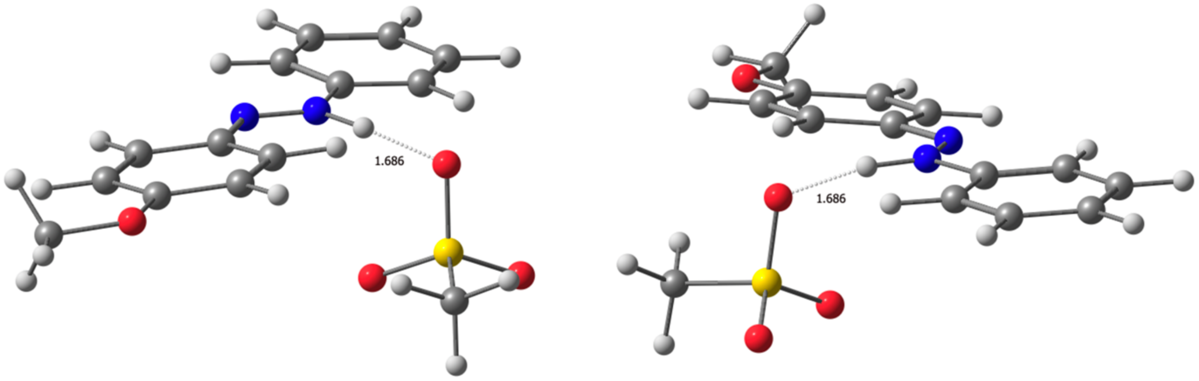

Figure S24: Geometry-optimized structure of ***2***H^+MSA^-.

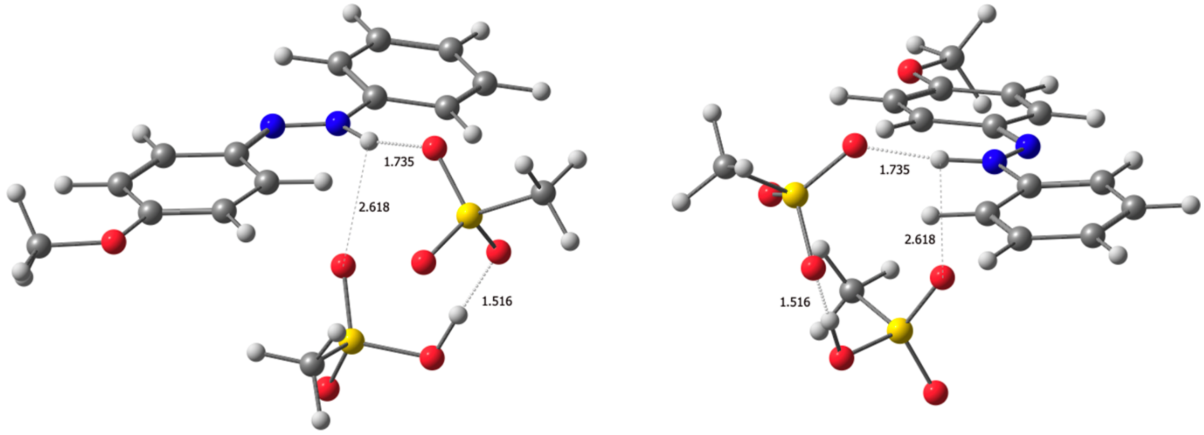

Figure S25: Geometry-optimized structure of ***2***H^+MSA^-MSA.

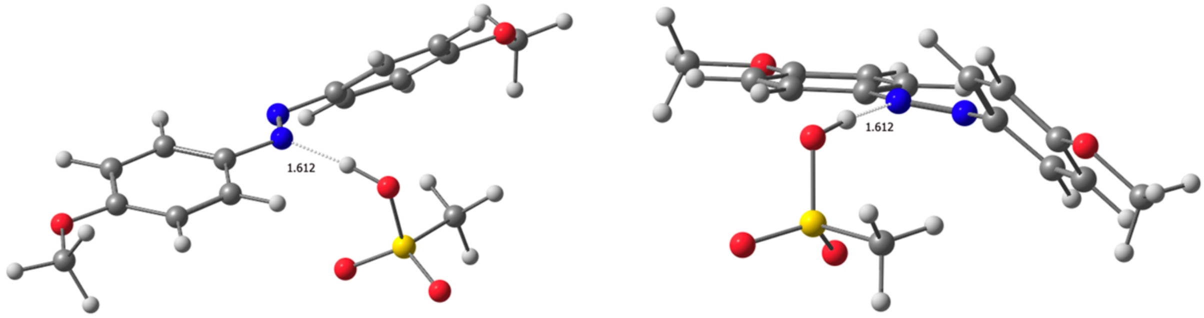

*Figure S26: Geometry-optimized structure of **3**-MSA.*

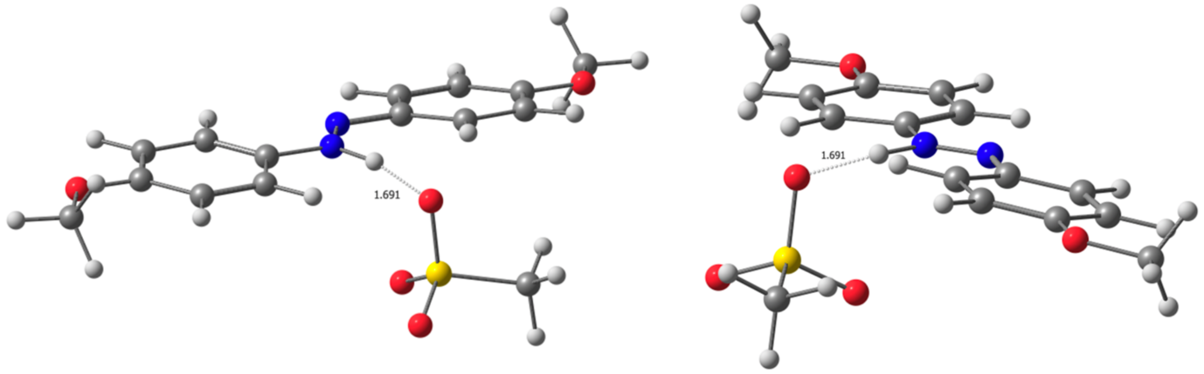

*Figure S27: Geometry-optimized structure of **3**H$^+$MSA$^-$.*

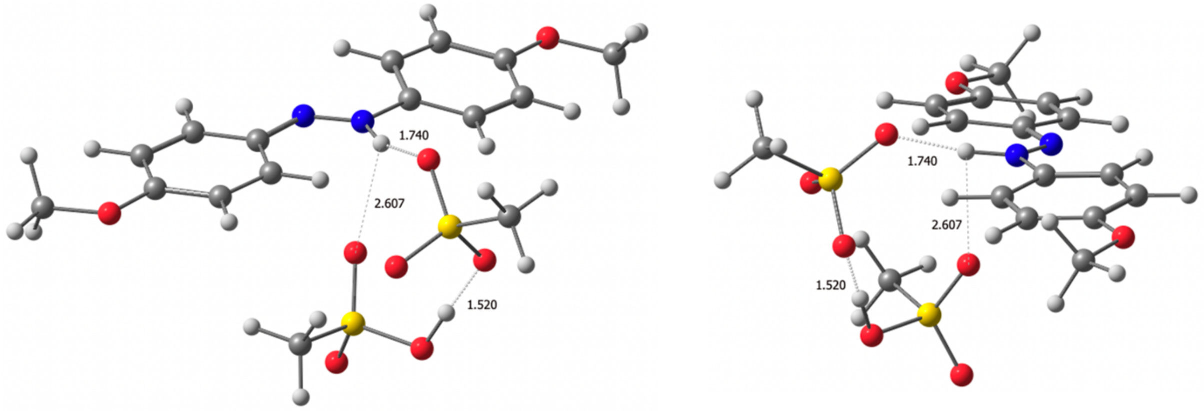

*Figure S28: Geometry-optimized structure of **3**H$^+$MSA$^-$MSA.*
